# CCAAT/Enhancer-Binding Protein *β* Mediates Oxygen-Induced Retinal Neovascularization via Retinal Vascular Damage and Vascular Endothelial Growth Factor

**DOI:** 10.1155/2020/2789209

**Published:** 2020-03-09

**Authors:** Tingting Li, Xuan Cai, Xiangning Wang, Xueyan Zhang, Hui Zhang, Biwei Xu, Shiwei Li, Jianyan Hu, Qiang Wu

**Affiliations:** Department of Ophthalmology, Shanghai Jiao Tong University Affiliated Sixth People's Hospital, Shanghai 200233, China

## Abstract

**Objective:**

To evaluate the role of CCAAT/enhancer-binding protein *β* (C/EBP *β* (C/EBP

**Methods:**

Rats with OIR were exposed to alternating hypoxic and hyperopic conditions for 14 days. Then, the rats with OIR were assigned randomly to groups that received intravitreal injections of either shRNA lentiviral particles targeting C/EBP *β* (C/EBP *β* (C/EBP *β* (C/EBP *β* (C/EBP *β* (C/EBP *β* (C/EBP

**Results:**

In OIR rats, the expression levels of C/EBP *β* (C/EBP *P* < 0.01). The p-C/EBP *β* (C/EBP *β* (C/EBP *β* (C/EBP *β* (C/EBP *β* (C/EBP *P* < 0.01). The p-C/EBP *β* (C/EBP *β* (C/EBP *β* (C/EBP *P* < 0.01). The p-C/EBP

**Conclusions:**

C/EBP *β* shRNA inhibits RNV in OIR. A potential mechanism may be that the activity of C/EBP *β* increases with its overexpression, which in turn aggravates the amount of the retinal vascular damage and promotes transcription of VEGF. C/EBP *β* might be a new therapeutic target for preventing RNV.*β* (C/EBP *β* (C/EBP *β* (C/EBP

## 1. Introduction

Retinal neovascularization (RNV) plays a crucial role in several common ocular diseases that cause blindness [1], such as proliferative diabetic retinopathy and retinopathy of prematurity. Retinal hypoxia caused by retinal vessel occlusion or capillary nonperfusion in retinopathies stimulates the production of angiogenic factors, leading to pathological vessel growth. These vessels are leaky and fragile, leading to fluid accumulation and haemorrhage, which results in damage to vision [2–4]. Accordingly, in recent years, various efforts have been made to investigate the mechanism of angiogenesis [5]. The findings are that different molecules, such as hypoxia-inducible factor (HIF), secretogranin III (Scg3), placental growth factor (PlGF), and vascular endothelial growth factor (VEGF), have shown the potential to be attractive therapeutic targets [6–9]. The primary growth factor associated with RNV is VEGF-A. However, some patients still have a poor response to anti-VEGF treatments, and alternative targeted treatments should be explored.

VEGF expression requires complex regulation of transcription, translation, and modification after translation. The step of transcription initiation, which is a complex process, is one of the important links in the process of gene regulation. During initiation, transcription factors become concentrated at promoter sequences to form a transcription initiation complex, which is associated with transcription initiation. Numerous studies have reported that many transcription factors in various tissues play roles in transcription and expression of VEGF including hypoxia-inducible factor- (HIF-) 1*α*, nuclear factor- (NF-) *κ*B, specificity protein 1 (Sp1), and activator protein-1 (AP-1). Our previous study used a luciferase assay and a chromatin immunoprecipitation assay (ChIP) to demonstrate that C/EBP *β* binds the VEGF-A promoter in the nucleus [10].

The C/EBP family of basic leucine-zipper (bZIP) transcription factors includes C/EBP *α*, *β*, *γ*, *δ*, and *ε*, as well as C/EBP homology protein (CHOP) [11]. C/EBP *β* was first identified as a nuclear protein that bound to an IL-1 response element in the IL-6 promoter region [12], and it was subsequently reported to regulate various genes involved in cell differentiation, proliferation, survival, and immune function and tumour invasiveness and progression [13–16]. Liu et al. [17] found that the interaction of C/EBP *β* with the IL-6 promoter maintained an angiogenic microenvironment and increased IL-6-driven angiogenesis. Huang et al. [18] showed that C/EBP *β* bound to the promoter region of VEGF-C to induce VEGF-C expression in IL-6-exposed lymphatic endothelial cells. Current research reports that C/EBP *β* binds to the VEGF promoter region and regulates *VEGF* expression in granulosa cells [19]. In the present study, we investigated a potential mechanism of RNV and demonstrated that C/EBP *β* mediates RNV in an oxygen-induced retinopathy (OIR) model.

## 2. Materials and Methods

### 2.1. OIR Animal Models and Intravitreal Injection Treatment

All animals were obtained from the Animal Laboratory of Shanghai Jiaotong University Affiliated Sixth People's Hospital. All procedures were performed in accordance with both the ARVO *Statement for the Use of Animals in Ophthalmic and Vision Research* and the *Guide for the Care and Use of Laboratory Animals* published by the National Institutes of Health. The protocols were approved by the Animal Ethics Committee of Shanghai Jiaotong University Affiliated Sixth People's Hospital.

The OIR model was induced according to a previously described method [10]. In brief, litters of Sprague-Dawley rat pups were placed in an alternate oxygen environment within 8 hours of birth and the oxygen level was cycled between 50% and 12% every 24 h for 14 days. The oxygen-exposed rats were returned to room air at postnatal day (P) 14 for up to 4 days, allowing time to develop RNV. Control rats were maintained in room air (RA) throughout the induction experiment.

A recombinant C/EBP *β* shRNA (ACAAGCTGAGCGACGAGTACA) and a scrambled shRNA were designed and packaged by GeneChem Co. Ltd. (Shanghai, China), as previously described [20]. At P8, the pups received an intravitreal injection with a 30-gauge needle and a syringe. For the present study, SD rats were divided into four groups: RA, normoxia control with room air conditions; OIR, OIR model rats without any treatment; OIR+LV.shScrambled, rats with OIR received intravitreal injection of 1 *μ*l control lentiviral particles; and OIR+LV.shC/EBP *β*, rats with OIR received intravitreal injection of 1 *μ*l 1 × 10^8^ TU/ml of C/EBP *β* shRNA lentiviral particles.

### 2.2. Fluorescein Isothiocyanate- (FITC-) Conjugated Dextran Retinal Angiography

The leakage of FITC-conjugated dextran from the retinal vasculature was assessed using the method of Zhang et al. [21]. Briefly, FITC-dextran (50 mg/ml in sterile PBS, Sigma Chemical Co., St. Louis, MO) was injected into the left ventricle on P14. Perfusion was considered successful if the mouth, nose, and external ear tissues turned yellow. The eyeballs were then enucleated and fixed in 4% PFA for at least 2 h in the room air. The peripheral retina was cut into 4 pieces and mounted with glycerol gelatine. The retinas were then viewed using a fluorescence microscope (Olympus, Tokyo, Japan). Images are presented at 20x magnification. The avascular area was measured using Photoshop CS4 software (Adobe, San Jose, CA).

### 2.3. Retinal Adenosine Diphosphatase (ADPase) Staining

Retinal vascular patterns were assessed by retinal ADPase staining at P18, as previously described [22]. This staining only marks retinal vascular endothelia and their stem cells in rats of this age [23]. The eyes were enucleated and fixed with 4% PFA for 3 h. The retinas were then dissected, flat-mounted after making 4 incisions in the centre of the disc, and processed for ADPase staining. The ADPase-stained retinas were then flat-mounted on microscope slides with a gelatine-coated cover slip and were carefully examined using an Olympus BH-2 microscope (McBain Instruments, Chatsworth, CA, USA). The local retinal images of the ADPase-stained retinas containing preretinal vessel tufts are presented at 50x magnification. The vessel tuft area was measured using Photoshop CS4 software (Adobe, San Jose, CA).

### 2.4. Trypsin Digestion and Periodic Acid-Schiff (PAS) Staining

Isolation of retinal vasculature and quantification of acellular capillaries were performed as previously described [24]. The enucleated eyes were fixed with 4% PFA for 24 h. The retinas were rinsed in distilled water overnight and then were incubated with 3% trypsin in 0.1 M Tris buffer (pH 7.8) at 37°C for 2 h. Nonvascular tissues were removed by gentle washing, and the isolated vasculature was flattened on poly-L-lysine-coated glass slides. After drying overnight, the retinal blood vessels were subjected to PAS and haematoxylin staining. Capillary networks were evaluated to identify the number of acellular capillaries. The numbers of endothelial cells and pericytes were determined by counting their respective nuclei under the microscope at 400x magnification. Six peripheral fields of retinal digestion from each retina were analysed for each retina in a blinded fashion. The average number of endothelial cells per mm^2^ vascular bed was calculated. The average ratio of endothelial cells to pericytes was used to demonstrate pericyte coverage or death.

### 2.5. Electroretinogram (ERG)

ERG measurements were performed according to previously published methods [25]. Before ERG testing, rats were dark adapted for a minimum of 16 h and then their manipulation was performed under dim red light. The animals were anaesthetized using 100 mg/kg ketamine and 10 mg/kg xylazine. Pupils were dilated with 0.5% atropine, and a heating pad was used to maintain the body temperature at 37.5°C. A subdermal ground electrode was inserted at the base of the tail, and the subdermal reference electrode was inserted at the forehead midway between the eyes. The ERG responses were stimulated by flashes of light ranging from −2 to 1 log cd·s/m^2^, which were generated by a Ganzfeld stimulator (Roland Consult, Brandenburg, Germany). The ERG response of both eyes was simultaneously recorded and analysed.

### 2.6. Immunohistochemistry

Eyes were fixed in 4% paraformaldehyde for 1 h, which was followed by incubation in 30% sucrose overnight. Retinal sections were blocked in 10% normal goat serum for 1 h at room temperature, which was followed by incubation with a C/EBP *β* monoclonal antibody (1 : 200, Cell Signaling Technology, Boston, MA, USA) overnight at 4°C. The retinal sections were incubated with HRP-conjugated secondary antibodies for 30 min at room temperature. Images were acquired using a 400x objective by an Olympus BH-2 microscope (McBain Instruments, Chatsworth, CA, USA).

### 2.7. Real-Time Polymerase Chain Reaction (RT-PCR)

Total RNA was extracted from retinas using the TRIzol reagent. RNA (2 *μ*g) was used for cDNA synthesis according to the manufacturer's instructions. The mRNA expression of C/EBP *β*, VEGF, and *β*-actin was quantified by quantitative real-time PCR. The mRNA level for each gene was normalized to *β*-actin mRNA. The primer sequences used were as follows: C/EBP *β* (forward: 5′-GGGTTGTTGCTGTTGATGTTTT-3′; reverse 5′-CTCGAAACGGAAAAGGTTCTC-3′), VEGF (forward: 5′-AAAGCCAGCACATAGGAGAG-3′; reverse: 5′-AGGATTTAAACCGGGATTTC-3′), and *β*-actin (forward: 5′-CACCCGCGAGTACAACCTTC-3′; reverse: 5′-CCCATACCCACCATCACACC-3′). Quantitative real-time PCR was performed using SYBR Green qPCR Super Mixture (Takara, Tokyo, Japan) and an ABI Prism 7500 Sequence Detection System. All reactions were performed in triplicate. The relative changes in gene expression were analysed by using the 2^-*△△*CT^ method.

### 2.8. Western Blot Analysis

Aliquots containing 30 *μ*g of protein were separated by SDS-polyacrylamide gel electrophoresis using a 10% gel, and the separated proteins were blotted onto polyvinylidene difluoride membranes (Millipore, Billerica, MA) in a wet transfer unit (Bio-Rad, Hercules, CA, USA). After blocking with 5% nonfat dry milk at room temperature for 1 h, the membranes were incubated overnight at 4°C with the following primary antibodies: anti-C/EBP *β* monoclonal antibody (1 : 1000, Cell Signaling Technology, Boston, MA, USA), anti-p-C/EBP *β* monoclonal antibody (1 : 1000, Abcam, Cambridge, MA, USA), anti-VEGF monoclonal antibody (1 : 200, Abcam, Cambridge, MA, USA), anti-histone H3 monoclonal antibody (1 : 1000, Proteintech Group, Chicago, IL), and anti-*β*-actin monoclonal antibody (1 : 5000, Sigma-Aldrich, Saint Louis, MO, USA). After being washed with TBS-Tween 20, the membranes were incubated with the appropriate HRP-conjugated secondary antibodies (1 : 1000, Proteintech Group, Chicago, IL) for 1 h at room temperature. The bands were visualized using an enhanced ECL detection kit (Bio-Rad, Hercules, CA).

### 2.9. Statistical Analysis

All data are presented as the mean ± standard deviation (SD). The data were analysed using SPSS 16.0 software. The difference between multiple groups was assessed by one-way ANOVA followed by Student-Newman-Keuls (SNK) comparisons. *P* < 0.05 was considered statistically significant.

## 3. Results

### 3.1. C/EBP *β* Expression Increased in the OIR Retinas and C/EBP *β* Was Primarily Located in the Ganglion Cell Layer (GCL) and the Inner Nuclear Layer (INL)

**Figure 1:**
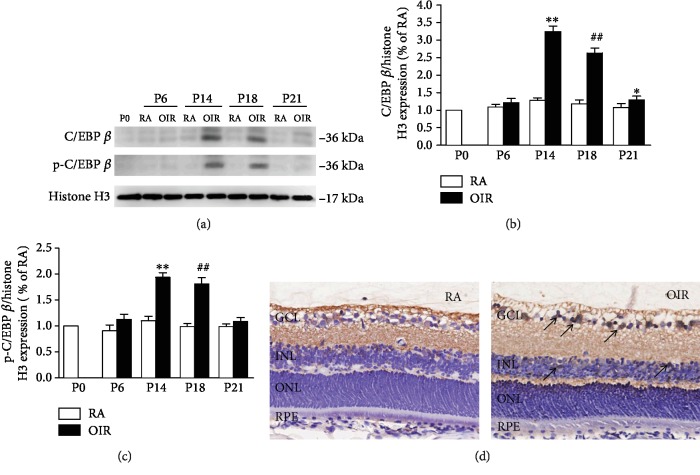
Temporal and spatial change of C/EBP *β* expression and the activity of C/EBP *β* in rats with OIR. (a) Expression of C/EBP *β* and p-C/EBP *β* protein (as determined by western blot) at different stage. (b) Quantitative analysis of the western blot signal density. Each column denotes the mean ± SD (*n* = 3). (c) Quantitative analysis of the western blot signal density. Each column denotes the mean ± SD (*n* = 3). (d) The location of C/EBP *β* in retinas from the control rats in room air conditions and the rats with OIR at P14. Black arrows denote the expression of C/EBP *β*. ^∗∗^*P* < 0.01 versus rats in the RA group at P14. ^##^*P* < 0.01 versus rats in the RA group at P18. ^∗^*P* < 0.05 versus rats in the RA group at P21. GCL: ganglion cell layer; INL: inner nuclear layer; ONL: outer nuclear layer; RPE: retinal pigment epithelium.

We first investigated the expression of C/EBP *β* in the OIR rats. The expression of C/EBP *β* was markedly increased in retinal samples from OIR rats at P14 and P18 compared with that of control RA rats, and the data displayed a time-dependent trend (*P* < 0.01, Figures 1(a) and 1(b)). Immunohistochemistry showed that C/EBP *β* was predominantly localized in the GCL and INL (Figure 1(d)). In addition, we also examined whether the expression of p-C/EBP *β* in the nucleus of the retinas increased with time and found the result to be consistent with the level of C/EBP *β* (*P* < 0.01, Figures 1(a) and 1(c)). We then investigated the effectiveness of transduction by performing intravitreal injection of C/EBP *β* shRNA. The retinal C/EBP *β* level was significantly reduced in tissues from rats transduced with LV.shC/EBP *β*-1 and LV.shC/EBP *β*-3 compared with tissues from those transduced with LV.shScrambled (*P* < 0.01, Supplemental [Supplementary-material supplementary-material-1]). However, there was no significant change in the C/EBP *β* level in tissues from rats transduced with LV.shC/EBP *β*-2 compared with tissues from those transduced with LV.shScrambled. Therefore, in later research, we used LV.shC/EBP *β*-1 to observe the role of C/EBP *β* in RNV.

### 3.2. C/EBP *β* shRNA Reduces the Oxygen-Induced Avascular Area in the Retina

**Figure 2:**
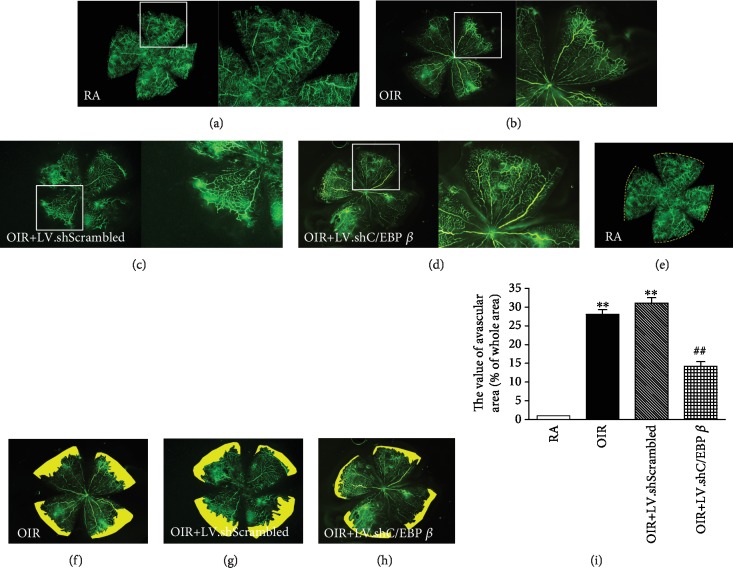
Retinal vascular patterns in flat mounts after FITC-dextran perfusion at P14. (a) Staining in retinas from the control rats in room air conditions. The white box indicates the boundary of the blood vessel and shows the corresponding magnification on the right. (b) Staining in retinas from rats with OIR without any treatment. The white box indicates the boundary of the blood vessel and shows the corresponding magnification on the right. (c) Staining in retinas from rats with OIR that received intravitreal injection of control lentiviral particles. The white box indicates the boundary of the blood vessel and shows the corresponding magnification on the right. (d) Staining in retinas from rats with OIR that received intravitreal injection of C/EBP *β* shRNA lentiviral particles. The white box indicates the boundary of the blood vessel and shows the corresponding magnification on the right. (e–h) Image produced in Photoshop. The avascular areas are yellow. (i) Quantitative analysis of the avascular areas. ^∗∗^*P* < 0.01 versus rats in the RA group. ^##^*P* < 0.01 versus rats in the LV.shScrambled group.

Vascular patterns were observed using FITC-dextran-perfused retinal flat mounts at P14 (Figure 2). The retinas from the RA group had superficial and deep vascular layers extending from the optic nerve to the periphery. These vessels formed a fine radial branching pattern in the superficial retinal layers and a polygonal reticular pattern in the deeper retinal layers. The retinas from the OIR group were characterized by having an avascular area mainly in the peripheral retina and a high degree of tortuosity of the major vessel (Figures 2(b) and 2(f)). The retinas from the OIR+LV.shScrambled group showed similar features as those of the OIR group retinas (Figures 2(c) and 2(g)). The retinas from the OIR+LV.shC/EBP *β* group exhibited partial attenuation of the retinal avascular area in the periphery (Figures 2(d), 2(h), and 2(i)). However, the degree of tortuosity of the major vessel was not different change in the retinas from the OIR+LV.shC/EBP *β* group.

### 3.3. C/EBP *β* shRNA Inhibits Oxygen-Induced Retinal Neovascularization

**Figure 3:**
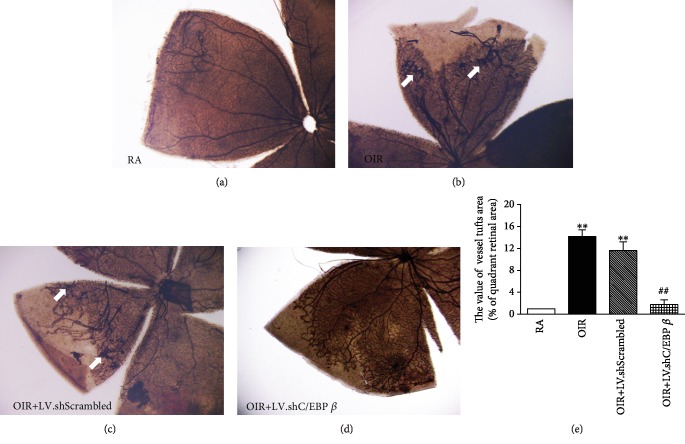
ADPase staining of peripheral retinas in flat mounts at P18. (a) Staining in retinas from the control rats in room air conditions. (b) Staining in retinas from rats with OIR without any treatment. White arrows denote abnormal neovascular tufts. (c) Staining in retinas from rats with OIR that received intravitreal injection of control lentiviral particles. White arrows denote abnormal neovascular tufts. (d) Staining in retinas from rats with OIR that received intravitreal injection of C/EBP *β* shRNA lentiviral particles. (e) Quantitative analysis of the vessel tuft area. ^∗∗^*P* < 0.01 versus rats in the RA group. ^##^*P* < 0.01 versus rats in the LV.shScrambled group.

ADPase staining was conducted to investigate the neovascular tufts in the retinas at P18 (Figure 3). Rats in the RA group showed no retinal pathology or avascular area at 18 days of age. The major vessels and capillaries were normal in shape (Figure 3(a)). Compared with the RA group, retinas from the OIR group revealed that abnormal preretinal neovascular tufts arose primarily at the peripheral-most extent of the major veins at high magnification. The major vessel distribution was disordered, and the capillary networks were destroyed (Figure 3(b)). Intravitreal injection with the C/EBP *β* shRNA lentivirus decreased abnormal neovascularization, whereas the LV.shScrambled had no such effect (Figures 3(c) and 3(d)). Quantitative analysis revealed that the neovascular tuft area was also reduced in the retinas from the OIR+LV.shC/EBP *β* group compared with those from the LV.shScrambled group (Figure 3(e)).

### 3.4. C/EBP *β* shRNA Attenuates Oxygen-Induced Loss of Retinal Pericytes

**Figure 4:**
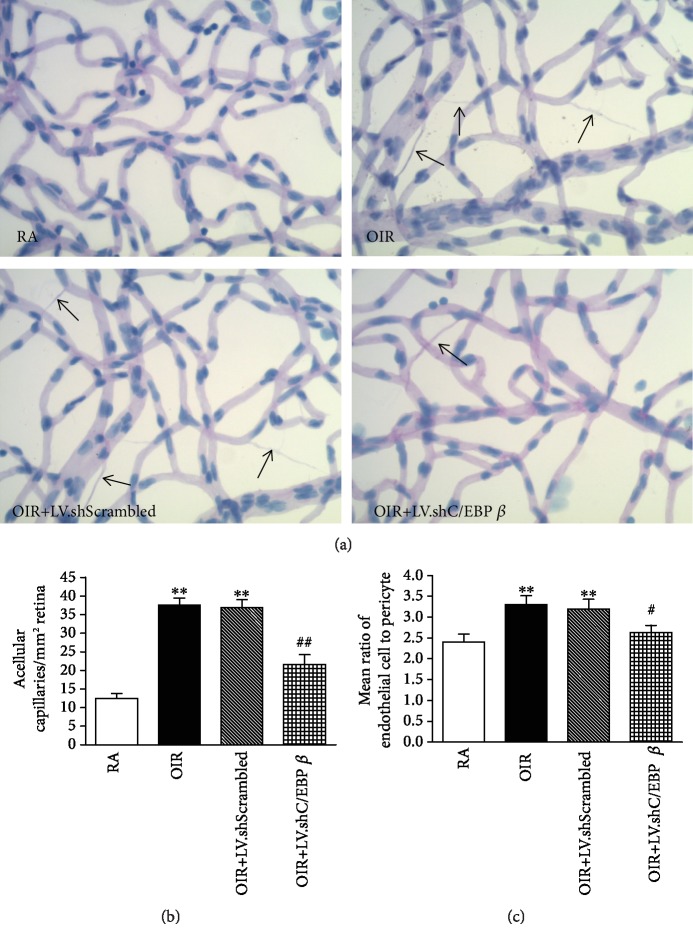
Trypsin-digested retinal blood vessels at P14. (a) Intact retinal vascular patterns. Black arrows denote the loss of pericytes and acellular capillaries. (b) Quantitative analysis of the mean number of acellular capillaries per mm^2^ retinal vasculature. (c) Quantitative analysis of the ratio of endothelial cells to pericytes. ^∗∗^*P* < 0.01 versus rats in the RA group. ^#^*P* < 0.05 versus rats in the LV.shScrambled group. ^##^*P* < 0.01 versus rats in the LV.shScrambled group.

Intact retinal vasculatures were used for quantitative analyses of acellular capillaries, endothelial cell density, and pericyte death (Figure 4). First, the number of acellular capillaries, which is indicative of capillary degeneration, was significantly increased in rats with OIR compared with what was observed in rats in the RA group and the mean number of acellular capillaries/mm^2^ in the retinal vasculature was significantly increased in the OIR group compared with the control group (*P* < 0.01, Figure 4(b)), but the C/EBP *β* shRNA lentivirus attenuated the OIR-induced capillary degeneration, which was not observed in the LV.shScrambled group (*P* < 0.01, Figure 4(b)). Second, pericyte death was significantly increased in rats with OIR compared with that of the RA control rats and the ratio of endothelial cells to pericytes was significantly increased in the OIR group compared with the control group (*P* < 0.01), but the C/EBP *β* shRNA lentivirus attenuated the OIR-induced pericyte loss to an extent not observed in the LV.shScrambled group (*P* < 0.05, Figure 4(c)).

### 3.5. C/EBP *β* shRNA Has no Effect on Oxygen-Induced Retinal Dysfunction

**Figure 5:**
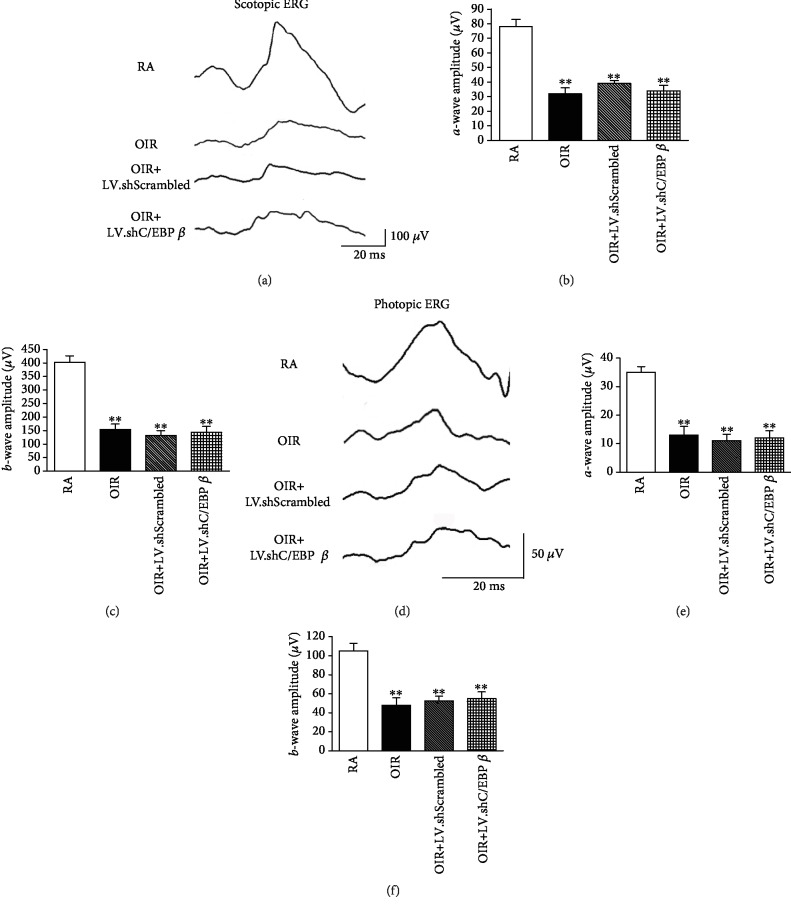
Effect of C/EBP *β* on the retinal dysfunction at P14. (a) Representative recordings of scotopic electroretinogram (ERG) in different rats. (b) Quantitative analysis of scotopic *a*-wave amplitudes. (c) Quantitative analysis of scotopic *b*-wave amplitudes. (d) Representative recordings of photopic ERG in different rats. (e) Quantitative analysis of photopic *a*-wave amplitudes. (f) Quantitative analysis of photopic *b*-wave amplitudes. ^∗∗^*P* < 0.01 versus rats in the RA group.

We determined whether the effects of C/EBP *β* on vascular injury were accompanied by retinal dysfunction by recording ERG responses to full-field light flashes. In the retinas at P14, the amplitudes of the *a*- and *b*-waves in the scotopic and photopic Ganzfeld ERG were significantly reduced in the rats with OIR compared with those of the rats in the RA group (Figure 5). Nevertheless, no differences in scotopic ERG and photopic ERG characteristics were detected between the rats of the LV.shC/EBP *β* group and those of the LV.shScrambled group (Figure 5).

### 3.6. C/EBP *β* shRNA Reduces VEGF Expression at the mRNA Level and Protein Levels

**Figure 6:**
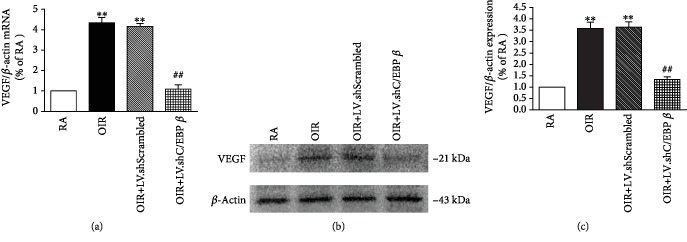
Effect of C/EBP *β* on the expression of VEGF at P18. (a) Expression of VEGF mRNA (as determined by RT-PCR). (b) Expression of VEGF protein (as determined by western blot). (c) Quantitative analysis of western blot signal density. Each column denotes the mean ± SD (*n* = 3). ^∗∗^*P* < 0.01 versus rats in the RA group. ^##^*P* < 0.01 versus rats in the LV.shScrambled group.

We sought to investigate the role of C/EBP *β* in regulating VEGF expression in the retinas of rats with OIR rat at P18. The results of RT-PCR and western blot analyses revealed that the mRNA and protein expression of VEGF was increased in the retinas of rats with OIR compared with that of the rats in the control RA group (*P* < 0.01), but the levels decreased significantly in the retinas from the LV.shC/EBP *β* group compared with those of the LV.shScrambled group (*P* < 0.01, Figure 6).

## 4. Discussion

In this study, C/EBP *β* was associated with the hypoxia-induced upregulation of VEGF expression and vascular damage in rats with OIR. C/EBP *β* is a transcription factor that is activated by phosphorylation and translocated to the nucleus subsequently. Microenvironmental changes caused by hypoxia are an important mechanism for regulating the expression of C/EBP *β*. In hypoxia-exposed periodontal ligament cells, C/EBP *β* mRNA expression increases significantly, which is dependent on the activity of the HIF-1 protein [26, 27]. Feng et al. [28] reported that intermittent hypoxia can significantly increase C/EBP *β* in human umbilical vein endothelial cells and can damage endothelial cells. Furthermore, Chen et al. [29] found that exposure of lung fibroblasts to hypoxia resulted in increased C/EBP *β* phosphorylation. Interestingly, an inverse correlation between the C/EBP *β* level and hypoxia stimulation was found in rheumatoid arthritis fibroblast-like synoviocytes [30]. Thus, changes in the level and activity of C/EBP *β* may play an important role in different tissues and organs. In our study, we also observed that the expression of C/EBP *β* and p-C/EBP *β* was increased in the retina of rats under hypoxic conditions, suggesting that the increase of p-C/EBP *β* was mainly due to the upregulation of the total C/EBP *β* expression. Our results support the hypothesis that reduced oxygen concentration mediates increased activity of C/EBP *β* through stimulating the overall level of C/EBP *β* in rats with OIR.

OIR is the major model used for the study of pathological angiogenesis, which occurs in most irreversible blinding eye diseases for all age groups, such as retinopathy of prematurity and proliferative diabetic retinopathy [31]. This model involves exposure to hyperoxia during early retinal development, leading to the arrest or retardation of normal retinal vascular development, which imitates the pathological characteristics of retinal neovascularization, with a consistent and reproducible angiogenic response [32]. Therefore, it has become a typical model in the investigation of the potential treatment for retinal neovascularization. In the present study, we chose the rat OIR model to evaluate the inhibitory effect of C/EBP *β* shRNA lentiviral particles on abnormal vascular changes in different phases. The rat model of OIR is more commonly used because it is economical and has low variability. Exposure to variable hyperoxia has been shown to be a much more effective stimulus of proliferative retinopathy in newborn rats than exposure to constant hyperoxia [23]. Penn et al. [23] found that the incidence of preretinal neovascularization was 97% in 50/10% rats and 72% in 80/40% rats. In our experiment, we raised rats in an environment cycling between 50% and 12% oxygen and found that the retinas from the OIR group had avascular areas primarily in the peripheral retina and showed a high degree of tortuosity of the major vessel using FITC-dextran-perfused retinal flat mounts on P14. Simultaneously, we examined the major vessel distribution disorder and capillary network destruction. Moreover, in the retinas at P18, abnormal preretinal neovascular tufts were observed at the peripheral-most extent of the major veins in the OIR group. Therefore, the 50/12% oxygen environment showed a high incidence rate of retinal vascular damage and pathological RNV, which provided a solid experimental foundation for our studies.

Previous reports have shown that C/EBP *β* is involved in neovascularization and vascular function. Zhang et al. [33] identified that miR-155 mimics could suppress choroidal neovascularization by inhibiting C/EBP *β* activity and M2 macrophage polarization. There was a report that C/EBP *β* depletion blocked EGFRvIII-mediated promotion of angiogenesis [17]. Another researcher showed that silencing C/EBP *β* could inhibit apoptosis to protect vascular endothelial cells from injury induced by intermittent hypoxia [28]. In our study, we observed that the increase of C/EBP *β* expression and activity was consistent with the pathological changes of OIR at P14 and P18. To observe the protective effect on retinal blood vessels, lentivirus-delivered C/EBP *β* shRNA was used, which inhibited the activity of C/EBP *β* through downregulation of the total level of C/EBP *β*. Our current results also demonstrated that the silencing of C/EBP *β* to some extent reduced hypoxia-induced retinal vessel damage containing the retinal avascular area, pericyte loss, and the ratio of endothelial cells to pericytes at P14 and further inhibited the formation of abnormal neovascular tufts at P18. In addition, retinal dysfunction also occurred in the OIR model by recording full-field ERG. The photoreceptor layer requires extraordinary oxygen to function properly, and it is presumed that as hypoxia-induced photoreceptors degenerate, their metabolic demands wane and the retinal vasculature becomes attenuated consequent to the neural retina's chronic decreased requirement for oxygen [34, 35]. However, there was no significant correlation between C/EBP *β* and retinal dysfunction. The improvement in vessel injury after recombinant shRNA-mediated inhibition of C/EBP *β* in our study adds further support to the idea that the activation of C/EBP *β* may accelerate the vascular damage course of ischemic retinopathy under hypoxic conditions. In ischemic retinopathies, a neovascular phase occurs subsequent to the initial vessel loss. Because avascular areas of the retina become hypoxic, this hypoxia induces the expression of angiogenic factors, such as VEGF, resulting in RNV. We conjecture that the reduction of neovascularization in the lentivirus-delivered C/EBP *β* shRNA-treated group may play somewhat of a role through regulation of vascular injury. Therefore, reducing the increase of C/EBP *β* activity caused by the overexpression of C/EBP *β* in the early stage of hypoxia may be an alternative to preventing and delaying vascular damage, which can inhibit pathological neovascularization in the retina.

VEGF is thought to be involved in most endothelial functions, including proliferation, migration, and angiogenesis. In this study, we compared the transcript and protein levels of the proangiogenic factor VEGF in the retinas of rats from the OIR and RA groups at P18 when we observed obviously abnormal neovascular tufts. The expression of VEGF increased in retinas in the OIR group compared with that in the RA retinas, and the difference was significant. Moreover, this trend of VEGF mRNA and protein was consistent with the upregulation of C/EBP *β* expression and activity at P18. This finding was consistent with other researches, showing that inactivation of C/EBP *β* prevented vasculogenesis in malignant tumours, possibly by regulating VEGF-mediated angiogenesis [17]. Furthermore, it was reported that VEGF regulated cancer cell motility and survival through activation of the C/EBP *β* pathway [36]. In uterine stromal cells, the reduction of C/EBP *β* mRNA levels led to a marked weak expression of VEGF [37, 38]. Lee et al. [36] indicated that C/EBP *β* was a downstream regulator of Tpl2 for CXCL1- or EGF-induced VEGF expression and the inactivation of C/EBP *β* was associated with downregulation of VEGF. In our previous research, we showed that C/EBP *β* bound the functional transcriptional factor binding site in the region of the VEGF promoter using luciferase reporter assays and a chromatin immunoprecipitation (ChIP) assay in retinal ganglion cells treated with 200 *μ*M CoCl_2_. The -115/-129, -893/-907, and -1304/-1318 regions in the 2000 bp promoter sequence upstream of the VEGF gene were most likely the potential binding sites for C/EBP *β* [10], which showed that C/EBP *β* was associated with the transcription initiation of VEGF. These results indicated that hypoxia instigated the C/EBP *β* expression, which in turn induced increase of C/EBP *β* activity and initiated binding of C/EBP *β* to the VEGF promoter and ultimately upregulated VEGF expression. Considering all these findings, we suggest that C/EBP *β* not only may induce RNV by inducing retinal injury but also may participate in RNV by regulating the expression of VEGF at the transcriptional level. The two modes of action may coregulate RNV in oxygen-induced retinopathy.

In the neovascularization process, vascular endothelial cells respond to alarm signals in the body by angiogenesis or inflammation. Inflammatory tissue is often hypoxic and can induce angiogenesis through upregulation of factors such as VEGF. Moreover, a previous report showed that VEGF induced the recruitment of leukocytes to the inflamed intestine, resulting in inflammation [39]. These results revealed that angiogenesis and chronic inflammation are codependent. It has also been reported that C/EBP *β* promotes the expression of the proinflammatory cytokines IL-1*β*, IL-6, and IL-8 to regulate inflammatory processes [17, 40, 41]. Thus, there is considerable evidence to suggest that C/EBP *β* might play a complicated role in pathological RNV, which is involved in the link between angiogenesis and inflammation.

In summary, intravitreal injection of a C/EBP *β* shRNA lentivirus successfully transduced rat retinas, resulting in the stable downregulation of C/EBP *β* expression and activity. C/EBP *β* shRNA inhibits retinal avascular and pericyte loss to further reduce RNV or inhibit RNV by modulating VEGF. Therefore, gene therapy with a lentivirus is a potentially effective treatment for RNV diseases; however, further investigations regarding the safety of lentiviruses in intravitreal injection are needed.

## Data Availability

The data used to support the findings of this study are available from the corresponding author upon request.

## Supplementary Materials

Supplementary Materials C/EBP *β* expression in rat retinas and lentiviral particle transduction. (A) Expression of C/EBP *β* mRNA (as determined by RT-PCR). (B) Expression of C/EBP *β* protein (as determined by western blot). (C) Quantitative analysis of the western blot signal density. Each column denotes the mean ± SD (*n* = 3). ^∗∗^*P* < 0.01 versus rats in the RA group. ^##^*P* < 0.01 versus rats in the LV.shScrambled group.
