# Supplementary material for: CCAAT/Enhancer-Binding Protein β Mediates Oxygen-Induced Retinal Neovascularization via Retinal Vascular Damage and Vascular Endothelial Growth Factor
Source: J Diabetes Res. 2020 Mar 9;2020:2789209. doi: 10.1155/2020/2789209 (PMC7085405; doi:10.1155/2020/2789209)
Supplement: Supplementary Materials — C/EBP β expression in rat retinas and lentiviral particle transduction. (A) Expression of C/EBP β mRNA (as determined by RT-PCR). (B) Expression of C/EBP β protein (as determined by western blot). (C) Quantitative analysis of the western blot signal density. Each column denotes the mean ± SD (n = 3). ∗∗P < 0.01 versus rats in the RA group. ##P < 0.01 versus rats in the LV.shScrambled group. [file 2789209.f1.pdf]

Materials and Methods

The sequences of three shC/EBP β. The recombinant C/EBP β shRNA (shRNA-1: ACAAGCTGAGCGACGAGTACA, shRNA-2: CATGGAAGTGGCCAACTTCTA, shRNA-3: CACGCTGCGGAACTTGTTCAA) and the scrambled shRNA were designed and packaged by Genechem Co., Ltd (Shanghai, China).

FIGURE CAPTIONS

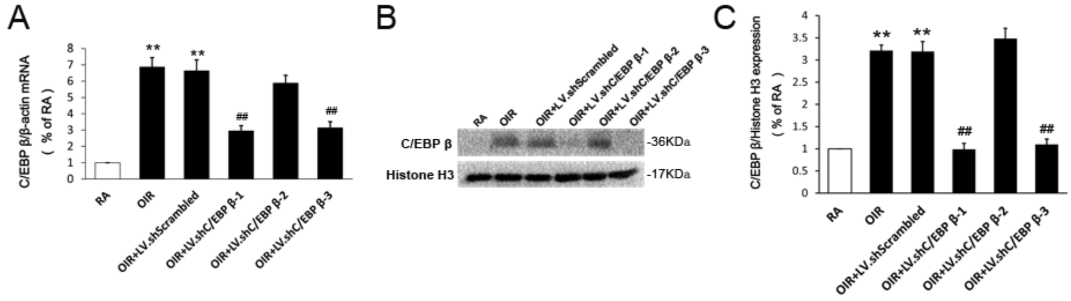

Supplemental Figure 1. C/EBP β expression in rat retinas and lentiviral particle transduction. (A) Expression of C/EBP β mRNA (as determined by RT-PCR). (B) Expression of C/EBP β protein (as determined by Western blot). (C) Quantitative analysis of the Western blot signal density. Each column denotes the mean ± SD (n=3). ***P*<0.01 versus rats in the RA group. ##*P*<0.01 versus rats in the LV.shScrambled group.
